# Comparison of the effect of lemborexant and other insomnia treatments on driving performance: a systematic review and meta-analysis

**DOI:** 10.1093/sleepadvances/zpab010

**Published:** 2021-07-03

**Authors:** Heather McElroy, Beth O’Leary, Michael Adena, Renee Campbell, Amir Abbas Tahami Monfared, Genevieve Meier

**Affiliations:** 1 Datalytics, Kingston ACT, Australia; 2 Formerly at Eisai Inc., Woodcliff Lake, NJ 07677, USA; 3 Eisai Inc., Woodcliff Lake, NJ 07677, USA

**Keywords:** driving performance, insomnia, meta-analysis, systematic literature review

## Abstract

**Study Objectives:**

This systematic literature review and meta-analysis explored the impact of lemborexant and other insomnia treatments on next-day driving performance.

**Methods:**

Searches were conducted in MEDLINE and Embase on May 16, 2019, supplemented by clinical trial registries. Randomized controlled trials in healthy volunteers or people with insomnia were included if they reported a standardized on-road driving test, were published in English and included ≥1 group receiving a recommended dose of flunitrazepam, estazolam, triazolam, temazepam, brotizolam, etizolam, alprazolam, lorazepam, zolpidem, eszopiclone, zaleplon, zopiclone, trazodone, ramelteon, lemborexant, or suvorexant. Pairwise random-effects meta-analyses used the difference between each active treatment and placebo in standard deviation of lateral position (ΔSDLP). ΔSDLP of +2.4 cm, established as equivalent to a blood alcohol concentration of 0.05%, was considered clinically significant.

**Results:**

Fourteen studies were included. Clinically significant differences in ΔSDLP were shown in healthy volunteers for zopiclone (10/10 studies) and ramelteon (1/1 study), and in people with insomnia for flunitrazepam (2/3 studies). Premature test termination was reported most frequently for zopiclone (5/10 studies) and was reported in two subjects for suvorexant (1/2 studies), one for flunitrazepam (1/3 studies), and one for placebo (1/12 studies). Lemborexant had no statistically or clinically significant ΔSDLP, and no premature driving test terminations.

**Conclusions:**

Zopiclone, flunitrazepam, and ramelteon were associated with impaired driving performance, similar to driving under the influence of alcohol. Premature test termination was reported most frequently for zopiclone, and also for suvorexant, flunitrazepam and placebo. Lemborexant had no statistically or clinically significant effect on driving performance.

**Statement of Significance**

Some insomnia treatments are associated with impaired next-day driving performance. This systematic literature review and meta-analysis explored the effect of lemborexant (a dual orexin receptor antagonist) and other insomnia treatments on next-day driving performance. Zopiclone, flunitrazepam and ramelteon were associated with impaired driving performance, similar to driving under the influence of alcohol. Premature test termination was reported for zopiclone, suvorexant, flunitrazepam and placebo. Lemborexant had no statistically or clinically significant change in standard deviation of lateral position relative to placebo (ΔSDLP), and no premature driving test terminations. This systematic review fills the gap in the literature by including next-day driving performance for lemborexant and suvorexant, which were not included in previous meta-analyses.

## Introduction

Insomnia is defined as difficulty in beginning or maintaining sleep or premature early-morning awakening, or a combination of these symptoms, with consequences for daytime functioning, when the symptoms are not attributable to environmental circumstances or lack of opportunity to sleep [1, 2]. Approximately one-third of the adult population in the United States suffers from insomnia, with more than 50% of the adult population self-reporting at least one of the insomnia symptoms [1, 3–6]. Insomnia is also a risk factor for unintentional fatal injuries including motor vehicle crashes, [7] workplace accidents and errors [5], and is associated with reduced quality of life [1, 8].

Current treatments for insomnia include non-pharmacological interventions such as cognitive behavioral therapy (CBT) and hypnotic drug treatments including benzodiazepines (BZDs), benzodiazepine receptor agonists (also called Z-drugs), melatonin agonists such as ramelteon, anti-depressants such as trazodone, and dual orexin receptor agonists (DORA) [1, 9]. Clinical practice guidelines for the treatment of insomnia in the United States [2] and Europe [9] recommend CBT as first-line treatment, with pharmacological therapy when CBT is unsuccessful or not available.

Next-day driving impairment is an important concern with the use of insomnia treatments, as indicated by studies showing an elevated risk of motor vehicle collisions in people treated with BZDs and Z-drugs [10], including older drivers [11]. The increased vehicle collision risk in people taking temazepam, trazodone or zolpidem is equivalent to the risk associated with blood alcohol concentrations of 0.06%–0.11%, which exceeds common legal driving limits of 0.05%–0.08% [12]. The prevalence of BZD use among drivers fatally injured in US motor vehicle crashes more than doubled between 1999–2000 and 2009–2010 [13], and 16.4% of fatally injured drivers aged 65 years or more tested positive for BZDs in an analysis of data from 14 US states in the period 2008–2012 [14]. Due to the residual sleepiness that can impair next-day functioning, physicians have consistently expressed reservations about using some of the current insomnia treatments, particularly BZDs and Z-drugs [1].

Lemborexant is a DORA recently approved for the treatment of insomnia in the United States [15], Japan, and Canada. The first DORA, suvorexant, was approved in the United States in 2014 [16]. The FDA’s guidance for industry called for “a systematic effort to identify drugs that increase the risk of motor vehicle accidents [as] a critical component of assessing drug risk and designing strategies to reduce this risk” [17]. Therefore, there is a need to update the literature with a meta-analysis of on-the-road driving studies for commonly prescribed insomnia medications with recent information available on new treatments, lemborexant and suvorexant, that were not included in previous meta-analyses. The objective of this systematic literature review and meta-analysis was to compare the effect of lemborexant and other treatments for insomnia on next-day driving performance in healthy volunteers and patients with insomnia, using published literature.

## Methods

### Search strategy

The primary objective of the literature review was to compare SDLP for lemborexant and other insomnia treatments in healthy volunteers, assessed by a standardized on-road driving test conducted the next morning, approximately 9–11 h after bedtime treatment. The secondary objectives were to compare SDLP in healthy volunteers and people with insomnia, and to describe the proportion of subjects who prematurely terminated the standardized on-road driving test for each treatment.

Literature searches of the Embase and Medline databases in Embase, and of PubMed to identify Epub Ahead of Print articles, were conducted on 16 May 2019. This was supplemented by a search of the International Clinical Trial Registry Portal (ICTRP) conducted on 7 June 2019. The Embase search strategy is provided in Table S1.

The eligibility criteria for study inclusion were based on Population, Intervention, Comparator, Outcome and Study design (PICOS) criteria as follows:

- Population: Healthy volunteers or adults with primary insomnia disorder;
- Intervention: At least one group randomized to one of 16 drug treatments: BZD (flunitrazepam, estazolam, triazolam, temazepam, brotizolam, etizolam, alprazolam, lorazepam), zolpidem (two formulations: controlled release [CR] and immediate release [IR]), eszopiclone, zaleplon, zopiclone, trazodone, ramelteon, lemborexant or suvorexant at doses recommended by Food and Drug Administration (FDA) in the United States, European Union 5 (EU5, France, Germany, Italy, Spain and United Kingdom) or Pharmaceutical and Medical Devices Agency (PMDA) in Japan. Combination treatments were excluded;
- Comparator: Other pharmacological treatments or placebo:
- Outcome: ΔSDLP measured in standardized on-road driving test, or premature termination of driving test;
- Study design: Randomized controlled trial (RCT).

There was no restriction on the timeframe of publication, and studies had to be published in English.

### Screening and data extraction

Studies identified in the search were screened by two independent reviewers, initially using the title and abstract, and then full text. Data were extracted by two independent reviewers, with differences resolved by consensus or by a third person. Extracted data included study characteristics (type of study, name of trial/study if applicable, selection criteria, design features, country, sample size), intervention and/or comparators assessed, and results (point estimate and measure of variability) for ΔSDLP and premature test terminations. Information for quality assessment was also extracted.

### Quality assessment

Quality of reporting for each study included in the review was assessed using the National Institute for Health and Care Excellence (NICE) methodology checklist for RCTs [18].

### Analysis

Standardized on-road driving tests have been developed to provide an objective assessment of the impact of drugs on driving ability [19]. Subjects are asked to drive for 100 km (50 km each way between fixed points) on a public highway in a specially instrumented vehicle, accompanied by a driving instructor with dual controls for safety reasons, while holding the vehicle steady within the slow lane at a constant speed. The primary outcome of the test is the standard deviation of lateral position (SDLP) during the test, which is assessed by continuously measuring the vehicle’s speed and lateral position within the lane. Other outcomes include premature termination of the test, either by the subjects (who are allowed to terminate the test if they feel unsafe to continue), or by the driving instructor (who can also terminate the test if he or she considers that the subject is driving unsafely). Benchmarks have been established to relate changes in SDLP relative to placebo (ΔSDLP) to the effect of specific levels of blood alcohol concentration. For example, a ΔSDLP of +2.4 cm is equivalent to a blood alcohol concentration of 0.05%, which is the legal limit for driving in many countries [20]. Zopiclone 7.5 mg has been shown to impair next-day driving performance by more than this benchmark and can be reliably used as a positive control in studies of driving performance [20].

**Figure 1.**
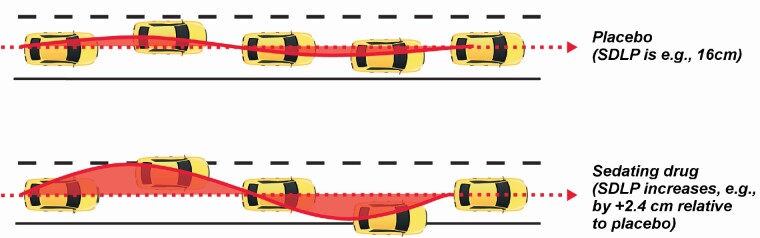
Measurement of standard deviation of lateral position (SDLP) in on-road driving test [21]. Reproduced with minor changes from: Vermeeren, A., Jongen, S., Murphy, P., Moline, M., Filippov, G., Pinner, K., Perdomo, C., Landry, I., Majid, O., Van Oers, A.C.M. *et al.* (2019). On-the-road driving performance the morning after bedtime administration of lemborexant in healthy adult and elderly volunteers. Sleep, 2019, 42 (4), 1–9, by permission of Oxford University Press (journal published on behalf of the Sleep Research Society). Diagrams are schematic and not to scale.

SDLP measures the degree of “weaving” or road tracking error displayed by the participant during the test. SDLP scores typically increase compared with placebo after ingestion of alcohol or hypnotic drugs, as illustrated in Figure 1 [21]. The meta-analysis considered the differences in SDLP between placebo and each active treatment (ΔSDLP).

The use of ΔSDLP as a proxy for safe driving performance was based on the well-established relationship between ΔSDLP and blood alcohol concentration [22]. Blood alcohol concentration of 0.05% equates to ΔSDLP of +2.4 cm, a blood alcohol concentration of 0.08% equates to ΔSDLP of +4.2 cm, and a blood alcohol concentration of 0.10% equates to ΔSDLP of +5.1 cm [23].

The analysis compared the difference between each active treatment and placebo. Pairwise random-effects meta-analyses were conducted in Stata version 15 using the mean and standard error of ΔSDLP reported for each crossover trial. If the standard error was not reported by the study authors, it was hand calculated from the confidence interval, *F*-statistic, or *p*-value.

The analysis was stratified into two groups, healthy volunteers and people with insomnia. This is because the magnitude of next-day residual sleepiness effects has been reported to be lower among people who regularly used BZD, zolpidem or zopiclone (at least 4 nights a week for 3 months) than in healthy controls [24]. It is therefore possible that studies in healthy volunteers could overestimate next-day impairment compared with studies in people with insomnia.

## Results

### Search results

**Figure 2.**
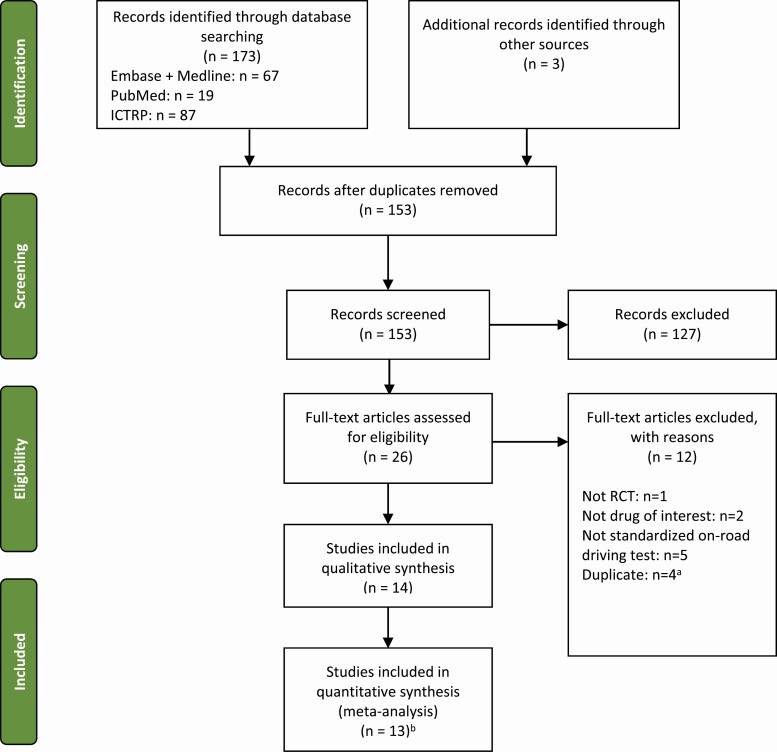
PRISMA flow diagram. ICTRP, International Clinical Trial Registry Portal; PRISMA, Preferred Reporting Items for Systematic Reviews and Meta Analyses. ^a^Four conference abstracts were excluded as the studies were later reported in a journal article. ^b^Results from O’Hanlon 1986 were not used in the meta-analysis.

The PRISMA flow diagram in Figure 2 summarizes the results of the literature searches and the screening/selection process. The database searches identified 173 articles, and three more were identified by manually by searching the reference lists of published reviews, making a total of 176 citations. Of these, 14 studies were included in the review [19, 21, 24–35].

One study [29] did not include a measure of variability and so was excluded from the meta-analysis, leaving a total of 13 studies included in the meta-analysis. Furthermore, the temazepam versus placebo comparison of interest in this study [29] was not randomized.

### Study characteristics

The characteristics of the included studies are summarized in Table 1. All were placebo-controlled crossover studies, and most also included an active control (typically zopiclone for more recent studies). Where reported, the washout period between different interventions was at least one week. All studies used a standardized on-road driving test 9–11 h after the bedtime administration of study drug, and most of the driving tests were conducted in the Netherlands. Publication dates ranged from 1984 to 2019.

**Table 1.** Summary of included studies

Trial ID	Interventions	Study design	Population	Number of subjects analyzed (number enrolled if different)	Timing of driving test
Leufkens 2009a [25]	ZOP 7.5 mg; PBO Excluded: Gaboxadol 15 mg (bedtime and MOTN); ZOL 10 mg (MOTN)	R, DB, PC, AC, five-way cross-over (washout NR)	Healthy volunteers – non elderly Aged 22–44 years BMI 19–29 kg/m^2^ Driving licence for >3 years and ≥5,000 km/year	25 (28)	10–11 h*
Leufkens 2009b [26]	TEM 20 mg; ZOP 7.5 mg; PBO	R, DB, PC, AC, three-way cross-over (washout NR)	Healthy volunteers – elderly Aged 55–75 years BMI 19–29 kg/m^2^ Driving ≥5,000 km/year over past 3 years	18	10–11 h
Leufkens 2014 [24]	ZOP 7.5 mg; PBO	DB, PC, 3 × 2 cross-over (washout ≥7 days)	Three groups: insomnia chronic users of hypnotics (BZD, ZOP or ZOL); insomnia infrequent users of hypnotics; good sleepers (matched for age and driving experience) Age 52–73 BMI 19–30 kg/m^2^ Driving ≥3,000 km/year over past 3 years	48 16 chronic users; 16 infrequent users; 16 good sleepers	10–11 h
Mets 2011 [27]	RAM 8mg; ZOP 7.5 mg; PBO	R, DB, PC, AC, three-way cross-over (washout ≥7 days)	Healthy volunteers – non elderly Age 21–55 years BMI 18-34kg/m^2^ Driving ≥5,000 km/year over past 3 years	30	8.5 or 10 h*
O’Hanlon 1984a [19]	FLUN 2 mg; PBO Excluded: loprazolam 1 mg; loprazolam 2 mg	DB, PC, four-way cross-over (washout NR)	Prior hypnotic users – non elderly Age 25–40 years Female Driving: owned and operated vehicle and drove ≥5,000 km/year SDLP ≤24 cm on training test	16	10–11.25 h
O’Hanlon 1984b [28]	FLUN 2 mg; ZOP 7.5 mg; PBO Excluded: nitrazepam 5 mg	DB, PC, cross-over (washout NR)	Prior hypnotic users Female	16	10 h
O’Hanlon 1986 [29]	TEM 20 mg; PBO Excluded: nitrazepam 10 mg	DB, 2-period active treatment cross-over (each period included SB PBO run-in and run-out before and after active treatment) (washout ≥14 days)	Insomnia and prior BZD – non elderly Female Driving: owned and operated own vehicle and ≥500 km/year for past 5 years	11 (12)	10–11.25 h
Ramaekers 2011 [30]	ZOP 7.5 mg; PBO Excluded: esmirtazapine 1.5 mg; esmirtazapine 4.5 mg	R, DB, PC, AC, four-way crossover (washout ≥7 days)	Healthy volunteers BMI 18–30 kg/m^2^ Driving: licence for >3yrs and ≥5,000 km/year	29 (32)	10–11.5 h
Vermeeren 1995 [31]	ZOL 10mg, FLUN 2 mg; PBO Excluded: partial sleep deprivation which was 1 week prior to drug component	DB, three-way cross-over (washout 7 days)	Insomnia and prior BZD – non elderly Female Age 25–51 years Driving: ≥5,000 km/year over past 3 years	17 (18)	10–11 h
Vermeeren 1998 [32]	ZAL 10 mg; ZOP 7.5 mg; PBO Excluded: ZAL 20 mg (bedtime and MOTN); ZAL 10 mg (MOTN); ZOP 7.5 mg (MOTN)	R, DB, PC, AC, seven-way cross-over (washout 7 days)	Healthy volunteers – non elderly Age 23–40 years Reported having driven between 8,000 and 60,000 km/year over past 3 years	28 (29)	10–11 h*
Vermeeren 2002 [33]	ZAL 10 mg; ZOP 7.5 mg; PBO Excluded: two-way PBO and alcohol cross-over	R, DB, PC, AC, three-way cross-over (washout ≥6 days)	Healthy volunteers – non elderly Age 21–45 years Driving: >5,000km/yr over past 3 years	30	10–11 h
Vermeeren 2015 [34]	SUV 20 mg; ZOP 7.5 mg; PBO Excluded: SUV 40mg	R, DB, PC, AC, four-way cross-over (washout ≥7 days)	Healthy volunteers – non elderly Aged 21–64 years BMI 19–30 kg/m^2^ Driving: ≥5,000 km/year over past 3 years	28	9 h
Vermeeren 2016 [35]	SUV 15 mg; ZOP 7.5 mg; PBO Excluded: SUV 30 mg	R, DB, PC, AC, four-way cross-over (washout ≥7 days)	Healthy volunteers – elderly Aged 65–80 years BMI 18–30 kg/m^2^ Driving: ≥3,000 km/year over past 3 years	24	9 h
Vermeeren 2019 [21]	LEM 5mg; LEM 10 mg; ZOP 7.5 mg; PBO Excluded: LEM 2.5 mg	R, DB, PC, AC, four-way incomplete cross-over (washout ≥14 days)	Healthy volunteers – non elderly and elderly Age 21 years and older BMI 18–30 kg/m^2^ Driving: ≥3,000 km/year over past 3 years	48 PBO, ZOP: 48 LEM doses: 32	9 h

AC, active control; BMI, body mass index; BZD, benzodiazepine; DB, double blind; FLUN, flunitrazepam; kg/m^2^; kilograms per metre-squared; km/yr, kilometres per year; LEM, lemborexant; mg, milligram; MOTN, middle of the night; PBO, placebo; PC, placebo control; NR, not reported; RAM, ramelteon; SUV, suvorexant; TEM, temazepam; yr, year; ZAL, zaleplon; ZOL, zolpidem immediate release; ZOP, zopiclone.

*Subjects were woken in the middle of the night to take placebo tablet (Leufkens 2009a, Vermeeren 1998) or to perform balance testing (Mets 2011).

Ten studies were conducted in healthy volunteers (including a group of self-defined good sleepers who did not meet any of the study criteria for insomnia and did not use hypnotics in Leufkens 2014 [24]) (Table 1). Five studies reported data in subjects with insomnia: two studies were in female subjects who had previously used hypnotic drugs [19, 28]; one enrolled subjects with acute insomnia treated with BZD in the previous 3–6 months [29]; one included subjects with chronic insomnia previously treated with a BZD, but who were hypnotic free for at least 2 weeks [31]; and one included subjects with insomnia for more than one month who were still using hypnotics frequently (chronic users) or infrequently (infrequent users) [24] (Table 1).

Seven studies investigated a single dose of insomnia treatment, [24–27, 31–33] two studies investigated BZDs and zopiclone over two consecutive nights [19, 28], and three studies investigated a DORA (lemborexant or suvorexant) over eight nights versus placebo, with zopiclone as an active control on the first and last night only [21, 34, 35]. The driving tests were conducted the morning after the first evening of study drug (Day 2) in 10 studies [21, 24–27, 31–35], or on the morning after the second evening of study drug (Day 3) in two studies [19, 28]. The 8-day studies also included a driving test on the morning following the eighth evening of study drug (Day 9). Drugs with long half-lives can result in markedly higher blood levels after multiple doses than occur after a single dose causing greater impairment with chronic use [17, 21, 34, 35].

The two sequences from Ramaekers 2011 [30] included in our review were (1) 7 days of placebo versus (2) 6 days of placebo followed by 1 day of zopiclone. The driving test following the first night of placebo in sequence (1) (placebo Day 2) was compared with the driving test following the first night of zopiclone (zopiclone Day 2, which is Day 8 of the sequence (2)).

The studies published before 1998 [19, 28, 29, 31] enrolled only female subjects (Table 1), while the more recent studies included men and women.

### Quality assessment

The quality assessment results are summarized in Table S2. In general, the studies were of high quality, following standard crossover designs. The older studies tended to be unclear about some aspects, such as randomization and an absence of conflict of interest information, and a few studies excluded subjects who discontinued early (Table 1).

### Standard deviation of lateral position relative to placebo (ΔSDLP) analysis

**Figure 3.**
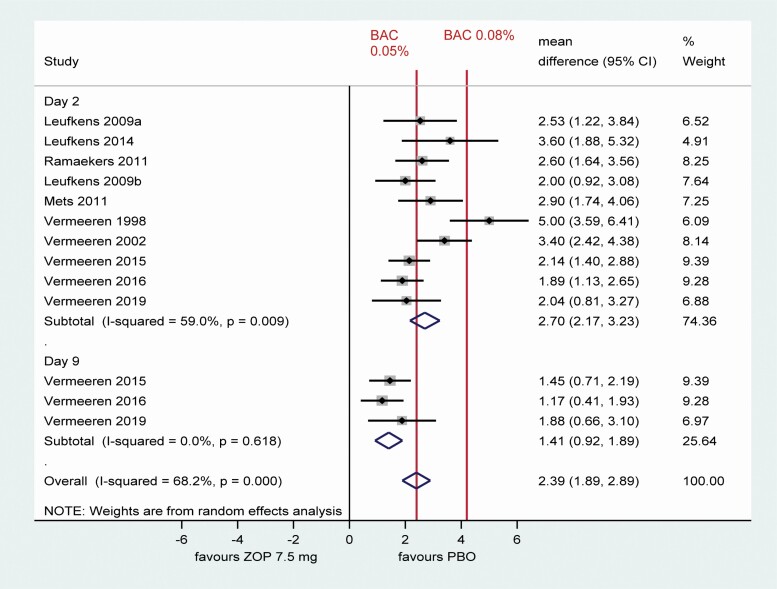
Pairwise random-effects meta-analysis of difference in SDLP between zopiclone and placebo in healthy volunteers. BAC, blood alcohol concentration; CI, confidence interval; PBO, placebo; ZOP, zopiclone. Note: Day 9 denotes driving test after ZOP on Day 1 and Day 8. Confidence intervals for individual studies may vary from those reported by study authors as they are based on z-statistic.

Clinically significant differences in ΔSDLP, with the 95% confidence interval (CI) crossing +2.4 cm (equivalent to driving with a blood alcohol concentration of 0.05%), were shown in healthy volunteers after a single evening dose of zopiclone 7.5 mg in all (10/10) studies (Figure 3). As zopiclone 7.5 mg acts as a positive control [20], this indicates that the ΔSDLP outcome measure was sensitive enough to detect driving impairment in all ten studies after a single evening dose. Of the three studies that also measured ΔSDLP after a second dose of zopiclone a week later (Day 9), the study of lemborexant [21] showed the same result at the second time point, whereas the two studies of suvorexant [34, 35] failed to demonstrate a clinically meaningful effect of zopiclone at the second time point as the 95% CI did not cross +2.4 cm (Figure 3).

**Figure 4.**
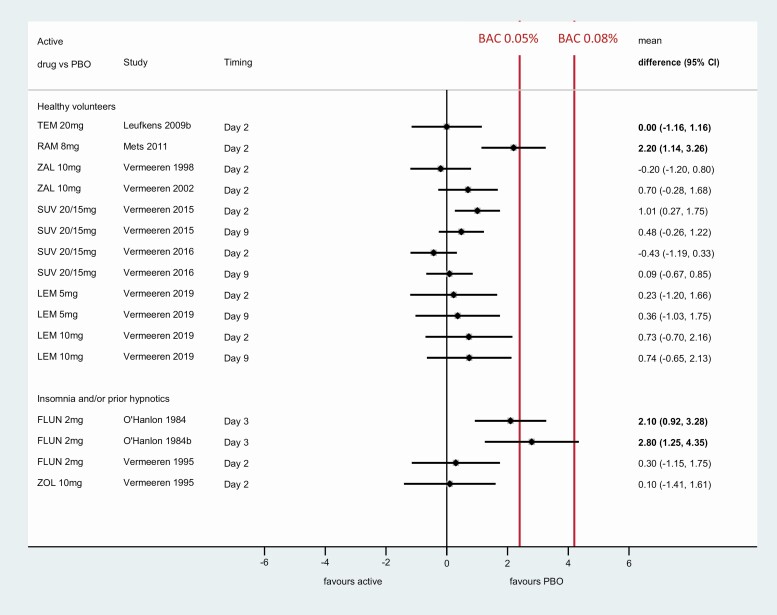
Pairwise difference in SDLP between active treatment and placebo in healthy volunteers and subjects with insomnia. BAC, blood alcohol concentration; CI, confidence interval; FLUN, flunitrazepam; LEM, lemborexant; PBO, placebo; RAM, ramelteon; SUV, suvorexant; TEM, temazepam; ZOL, zolpidem immediate release; ZAL, zaleplon. Note: Day 2 denoted driving test following single bedtime administration of active treatment or placebo; Day 3 denotes driving test after 2 days of bedtime administration of active treatment and Day 9 denotes driving test following 8 days of bedtime administration of active treatment. Confidence intervals for individual studies may vary from those reported by study authors as they are based on z-statistic.

Figure 4 shows meta-analysis results for ΔSDLP for treatments other than zopiclone in single or multiple doses, including healthy volunteers and subjects with insomnia or prior use of hypnotics.

Flunitrazepam 2 mg was only studied in patients with insomnia and showed clinically meaningful changes in ΔSDLP in two out of three studies (Figure 4). The other treatment studied in patients with insomnia, zolpidem IR, showed no clinically meaningful effect on ΔSDLP (Figure 4).

Lemborexant 5 mg or 10 mg showed no statistically or clinically significant difference from placebo in SDLP after a single dose, or after 8 days of treatment, in healthy volunteers (Figure 4). Suvorexant also showed no clinically meaningful effect on ΔSDLP after a single dose or after 8 days of treatment in healthy volunteers, as was also the case for zaleplon and temazepam after a single dose (Figure 4). Ramelteon was the only treatment that showed a clinically meaningful change in ΔSDLP after a single dose in healthy volunteers (Figure 4).

### Premature termination of driving test

Results for premature termination of the driving test are summarized in Table 2. Two studies did not report data. Premature termination of the driving test was reported most frequently for zopiclone 7.5 mg. At least one test was terminated in four out of nine studies of zopiclone in healthy volunteers and in one of the two studies (in the infrequent hypnotic user group) in people with insomnia (Table 2). This is consistent with the established effects of zopiclone on next-day driving performance.

**Table 2.** Premature termination of driving test

Trial ID	Test day	PBO	ZOP	LEM	SUV	ZOL	ZAL	FLUN	TEM	RAM
**Healthy volunteers**										
Leufkens 2009a [25]	Day 2	0/25	0/25							
Leufkens 2009b [26]	Day 2	0/18	1/18 (6%)*						0/18	
Leufkens 2014 [24]	Day 2	0/16	0/16							
Mets 2011 [27]	Day 2	0/30	0/30							0/30
Ramaekers 2011 [30]	Day 2	NR	NR							
Vermeeren 1998 [32]	Day 2	0/28	2/28 (7%)†				0/28			
Vermeeren 2002 [33]	Day 2	0/30	1/30 (3%)*				0/30			
Vermeeren 2015 [34]	Day 2	0/28	0/28		1/28 (4%)‡					
	Day 9	0/28	0/28		1/28 (4%)‡					
Vermeeren 2016 [35]	Day 2	1/24 (4%)*	0/24		0/24					
	Day 9	0/24	0/24		0/24					
Vermeeren 2019 [21]	Day 2	0/48	1/48 (2%)‡	5mg: 0/32 10mg: 0/32						
	Day 9	0/48	2/48 (4%)†	5mg: 0/32 10mg: 0/32						
**Insomnia**										
Leufkens 2014 – CU [24]	Day 2	0/16	0/16							
Leufkens 2014 – IU [24]	Day 2	0/16	1/16 (6%)*							
O’Hanlon 1984a [19]	Day 3	0/16						0/16		
O’Hanlon 1984b [28]	Day 3	0/16	0/16					0/16		
O’Hanlon 1986 [29]	Day 3	NR							NR	
Vermeeren 1995 [31]	Day 2	0/17				0/17		1/17 (6%)*		

CU, chronic hypnotic users; FLUN, flunitrazepam; IU, infrequent hypnotic users; LEM, lemborexant; NR, not reported; PBO, placebo; RAM, ramelteon; SUV, suvorexant; TEM, temazepam; ZAL, zaleplon; ZOL, zolpidem; ZOP, zopiclone.

*Test terminated by the instructor.

^†^One test stopped by the subject and one stopped by the instructor.

^‡^Test terminated by the subject.

Premature test termination was reported for one subject with insomnia after flunitrazepam in one of the three studies of flunitrazepam (all in people with insomnia) (Table 2). The instructor also terminated the test in one healthy volunteer after a single dose of placebo in one study of the nine studies that reported premature test termination data in healthy volunteers; there were no premature terminations reported after placebo in people with insomnia (Table 2).

While on-the-road driving studies for suvorexant 15 mg and 20 mg demonstrated no clinically significant impairment in next-morning driving performance, some individuals in the study had to stop driving tests prematurely due to somnolence [34]. One healthy volunteer terminated the test because they felt too drowsy to continue driving following suvorexant 20 mg on Day 2, and another did the same on Day 9 in one of the two studies reporting data on suvorexant [34] (Table 2). None of the healthy volunteers taking lemborexant had a premature driving test termination, either after a single dose or 8 days of treatment (Table 2). No studies investigated driving performance after lemborexant or suvorexant in people with insomnia. No premature terminations of driving tests were reported for temazepam, ramelteon, zaleplon or zolpidem IR (Table 2).

## Discussion

This review and meta-analysis examined evidence on the effect of lemborexant compared with other insomnia treatments on next-day driving performance across 14 studies, 13 of which were included in the meta-analysis. To our knowledge, this is the first systematic literature review and meta-analysis on the effect of insomnia treatments on next-day driving performance to include lemborexant and suvorexant.

All ten studies in healthy volunteers reported that zopiclone 7.5 mg had a clinically meaningful effect on ΔSDLP, equivalent to a blood alcohol level of 0.05%, demonstrating the sensitivity of the ΔSDLP measure in the studies [20]. Ramelteon was the only treatment to show clinically significant effects on ΔSDLP in healthy volunteers, reported in one study. Zaleplon, temazepam and suvorexant showed no clinically meaningful impairment of ΔSDLP after a single dose in healthy volunteers, and suvorexant also showed no clinically meaningful impairment of ΔSDLP after 8 days of treatment. Flunitrazepam was associated with clinically significant effects on ΔSDLP in two out of three studies in people with insomnia (no flunitrazepam studies were conducted in healthy volunteers). Lemborexant 5 mg or 10 mg showed no impairment of driving performance, as measured by ΔSDLP after a single dose or after 8 days of treatment. Furthermore, no subject receiving lemborexant terminated the driving test early.

Our results are consistent with previous meta-analyses of treatments for insomnia on driving performance. Given that these previous analyses did not include DORAs, only comparisons of other insomnia treatments could be made between this present study and previous meta-analyses. Two previous meta-analyses found that BZDs overall and zopiclone impaired driving the morning after a bedtime dose, while zolpidem and zaleplon did not [36, 37], consistent with our findings in the present study, although we analyzed the BZDs, flunitrazepam and temazepam, separately. A review of experimental studies and roadside evidence concluded that zolpidem taken before 8 h of uninterrupted sleep is a safe alternative to BZDs and zopiclone, which show significant driving impairment the morning after bedtime administration [38]. BZDs were associated with significant driving impairment in a meta-analysis of four publications using SDLP as the outcome measure [39], similar to our findings with the BZD flunitrazepam in the present study. A meta-analysis of 8 studies of zopiclone using ΔSDLP as the outcome measure found significant next-day driving impairment, supporting the use of zopiclone as a positive control in on-road driving tests as well as in the present meta-analysis [20].

A recent meta-analysis of insomnia treatments published in 2020 compared lemborexant and suvorexant but did not cover outcomes related to driving performance [16]. Another meta-analysis of insomnia treatments by the present authors, currently in press, covered a range of insomnia treatments including lemborexant and suvorexant, but did not cover driving outcomes [40].

The FDA recommend a starting dose for suvorexant of 10 mg, not exceeding 20 mg [41], and there is a warning against next-day driving for the 20 mg dose [42]. Likewise, the FDA recommended a starting dose of lemborexant 5 mg, and there is a warning against next-day driving for the 10mg dose [43]. However, lemborexant at doses of 1–10 mg has previously been shown to have no significant effect compared with placebo on subjective residual morning sleepiness as measured by the Karolinska Sleepiness Scale, and no consistent effect compared with placebo on objective measures of reaction time [44], which is consistent with the absence of effect on next-day driving performance. Lemborexant has been shown to not have significant residual morning effects as demonstrated by the change from baseline in postural stability not being different from placebo, sleep diary entries showing more alertness with lemborexant versus placebo after 6 months of treatment, and no significant impairment of driving performance versus placebo [45]. It should be noted that there are cautions about next-day driving performance in the FDA-approved labeling for zaleplon [46], temazepam [47] and higher doses of suvorexant [42] and lemborexant [43], although our analysis found no evidence of clinically meaningful changes in ΔSDLP for these agents. This is because the FDA considers multiple strands of evidence in relation to driving tests when deciding on labeling.

Future studies conducted in insomnia patients may be helpful in exploring the interaction between residual next-day treatment effects and the impact of insomnia itself, other comorbid conditions and other concomitant medications on driving performance. Apart from the use of zopiclone as a positive control, the studies identified in this review did not directly compare between the active treatments of interest. The meta-analysis focused on comparisons between each active treatment and placebo, rather than differences between active treatments. Studies comparing active insomnia treatments, and studies of the effect of long-term treatment, could be valuable areas for future research.

### Limitations

The studies included in the review were published over a wide date range of 35 years (1984–2019). This could potentially introduce limitations if there were changes in study methodology over such a wide date range. However, the studies included for the primary outcome measure analyzed (ΔSDLP in healthy volunteers) were conducted using a highly standardized test and mainly by the same research group in the Netherlands. Consequently, minimal heterogeneity would be expected.

Only outcomes from standardized on-road driving tests (ΔSDLP and premature test termination) were included in the review. Other possible outcome measures such as attention lapses and tests that are not conducted on-road such as those using driving simulators exist, but these outcomes were not the focus of this review and are not consistently/widely reported. However, ΔSDLP in a standardized on-road driving test is a well-established measure of driving performance that has been used for decades and was also an endpoint commonly used in the studies included in the present meta-analysis. As noted above, use of this standardized outcome measure may have helped minimize heterogeneity in methodology despite the wide date range of the studies included.

The evidence base revealed by the searches appears to be limited and patchy. Published evidence was found for only 8 of the 16 treatments in the search (zopiclone, zolpidem, zaleplon, lemborexant, suvorexant, flunitrazepam, temazepam and ramelteon). No on-road driving studies measuring SDLP were identified for estazolam, triazolam, brotizolam, etizolam, alprazolam, lorazepam, eszopiclone or trazodone. Only three studies of two interventions (lemborexant and suvorexant) investigated treatment duration of 8 days, whereas all the other studies investigated treatment for one or two nights only. No studies were identified with treatment durations of more than 8 days, indicating a lack of data on the effects of long-term insomnia treatment.

Premature termination of the driving test is a subjective measure, and is not necessarily related to objectively impaired driving performance, particularly when the decision to stop is made by the subject rather than the instructor. Subjects may decide to stop driving if they are aware of or concerned about potential drug effects on performance [34].

It could also be considered a limitation that most of the studies identified were conducted in healthy volunteers rather than in people with insomnia. However, the use of healthy volunteers is the norm in on-road assessments of driving performance [34]. Furthermore, there is evidence that healthy volunteers are more sensitive to the next-day effects of sedative drugs than patients with insomnia [24]. Conducting studies of driving performance in healthy volunteers therefore minimizes the risk of failing to detect clinically relevant next-day impairment.

## Conclusions

In this systematic review of 14 studies investigating next-day driving performance in healthy volunteers and patients with insomnia receiving a range of insomnia treatments, zopiclone, flunitrazepam and ramelteon were associated with impaired driving performance, similar to driving under the influence of alcohol. Premature test termination was reported most frequently for zopiclone, and also for suvorexant, flunitrazepam and placebo. Lemborexant 5 mg or 10 mg had no statistically or clinically significant effect on next-day driving performance, and no premature terminations of the driving test. Lemborexant is an effective treatment for insomnia that does not impair next-day driving performance compared to other insomnia medications.

## Supplementary Material

zpab010_suppl_Supplementary_Materials
