# Supplementary material for: Comparison of the effect of lemborexant and other insomnia treatments on driving performance: a systematic review and meta-analysis
Source: Sleep Adv. 2021 Jul 3;2(1):zpab010. doi: 10.1093/sleepadvances/zpab010 (PMC10104353; doi:10.1093/sleepadvances/zpab010)
Supplement: zpab010_suppl_Supplementary_Materials [file zpab010_suppl_supplementary_materials.docx]

**Supplementary material**

**List of supplementary material captions**

**Table S1. Embase search strategy**

**Table S2. Summary of quality assessment**

**Table S1. Embase search strategy**

| No. | Query |
| --- | --- |
| #63 | #62 AND [english]/lim |
| #62 | #53 OR #61 |
| #61 | #55 AND #57 AND #60 |
| #60 | #51 OR #59 |
| #59 | 'standard deviation of lateral position':ab OR sdlp:ab |
| #58 | #56 OR #57 |
| #57 | zaleplon:ab OR zolpidem:ab OR zopliclone:ab OR eszopliclone:ab OR ramelteon:ab OR triazolam:ab OR temazepam:ab OR lorazepam:ab OR flunitrazepam:ab OR brotizolam:ab OR estazolam:ab OR etizolam:ab OR alprazolam:ab OR suvorexant:ab OR lemborexant:ab OR trazodone:ab OR 'sleep medication':ab |
| #56 | 'sleep medicine'/mj |
| #55 | #4 OR #5 OR #6 OR #54 |
| #54 | 'general population':ab |
| #53 | #7 AND #46 AND #52 |
| #52 | #47 OR #48 OR #49 OR #50 OR #51 |
| #51 | 'standard deviation of lateral position':de |
| #50 | driving:ti OR driver:ti OR 'on-the-road':ti |
| #49 | 'highway driving test':de OR 'next morning on the road driving performance':de |
| #48 | 'car driving'/mj |
| #47 | 'driving ability'/mj |
| #46 | #8 OR #9 OR #10 OR #11 OR #12 OR #13 OR #14 OR #15 OR #16 OR #17 OR #18 OR #19 OR #20 OR #21 OR #22 OR #23 OR #24 OR #25 OR #26 OR #27 OR #28 OR #29 OR #30 OR #31 OR #32 OR #33 OR #34 OR #35 OR #36 OR #37 OR #38 OR #39 OR #40 OR #41 OR #42 OR #43 OR #44 OR #45 |
| #45 | trazodone:ti OR oleptro:ti OR desyrel:ti OR act541468:ti OR 'act 541468':ti |
| #44 | 'trazodone'/mj |
| #43 | lemborexant:ti OR 'e 2006':ti OR e2006:ti OR 'lem 10':ti OR 'lem 5':ti |
| #42 | 'lemborexant'/mj |
| #41 | suvorexant:ti OR 'mk 4305':ti OR mk4305:ti |
| #40 | 'suvorexant'/mj |
| #39 | alprazolam:ti OR xanax:ti OR alprax:ti OR kalma:ti |
| #38 | 'alprazolam'/mj |
| #37 | etizolam:ti OR depas:ti OR etilaam:ti OR etizest:ti OR etizola:ti OR pasaden:ti OR sedekopan:ti |
| #36 | 'etizolam'/mj |
| #35 | estazolam:ti OR prosom:ti OR eurodin:ti |
| #34 | 'estazolam'/mj |
| #33 | brotizolam:ti OR bondormin:ti OR lendormin:ti OR we941:ti OR 'we 941':ti |
| #32 | 'brotizolam'/mj |
| #31 | flunitrazepam:ti OR rohypnol:ti OR hypnodorm:ti |
| #30 | 'flunitrazepam'/mj |
| #29 | lorazepam:ti OR ativan:ti OR temesta:ti OR subveta:ti OR truveta:ti OR 'edg 004':ti |
| #28 | 'lorazepam'/mj |
| #27 | temazepam:ti OR temaze:ti OR temtabs:ti OR normison:ti OR restoril:ti |
| #26 | 'temazepam'/mj |
| #25 | triazolam:ti OR halcion:ti OR hypnostat:ti OR tgar01h:ti |
| #24 | 'triazolam'/mj |
| #23 | ramelteon:ti OR rozerem:ti OR 'tak 375':ti OR tak375:ti |
| #22 | 'ramelteon'/mj |
| #21 | eszopiclone:ti OR lunesta:ti OR 's zopiclone':ti OR esopiclone:ti OR estorra:ti OR lunivia:ti |
| #20 | 'eszopiclone'/mj |
| #19 | zileze:ti OR zimoclone:ti OR zimovane:ti OR 'zopi puren':ti OR zopicalm:ti OR zopicalma:ti OR zopiclodura:ti OR zopiclon:ti OR zopitan:ti OR zoplicon:ti OR zorclone:ti |
| #18 | zopiclone:ti OR amoban:ti OR imovance:ti OR imovane:ti OR limovan:ti OR optidorm:ti OR rhovane:ti OR 'rp 27267':ti OR siaten:ti OR somnosan:ti OR ximovan:ti |
| #17 | 'zopiclone'/mj |
| #16 | ziohex:ti OR zodorm:ti OR zodormdura:ti OR zoldem:ti OR zolirin:ti OR 'zolpi lich':ti OR zolpimist:ti OR zolpinox:ti OR zopidem:ti OR zopim:ti |
| #15 | 'sl 800750 23n':ti OR somit:ti OR somnil:ti OR somno:ti OR stilnix:ti OR stilnoct:ti OR stilnox:ti OR stilpidem:ti OR supedal:ti OR 'tovalt odt':ti |
| #14 | 'zolpidem tartrate':ti OR ambien:ti OR adormix:ti OR amsic:ti OR bikalm:ti OR dalparan:ti OR durnit:ti OR edluar:ti OR myslee:ti OR niotal:ti OR nitrest:ti |
| #13 | 'zolpidem tartrate'/mj |
| #12 | zolpidem:ti OR 'sl 800750':ti OR sl800750:ti |
| #11 | 'zolpidem'/mj |
| #10 | 'cl 284846':ti OR cl284846:ti OR 'l 846':ti OR l846:ti OR 'ljc 10846':ti OR ljc10846:ti OR 'skp 1041':ti |
| #9 | zaleplon:ti OR sonata:ti OR hegon:ti OR hipnodem:ti OR noctiplon:ti OR plenidon:ti OR starnoc:ti OR zaplon:ti OR zelepion:ti OR zerene:ti |
| #8 | 'zaleplon'/mj |
| #7 | #1 OR #2 OR #3 OR #4 OR #5 OR #6 |
| #6 | 'healthy adult':ti OR 'healthy adults':ti OR 'healthy elderly':ti OR 'healthy subjects':ti OR 'healthy volunteers':ti |
| #5 | 'human experiment'/de |
| #4 | 'normal human'/de |
| #3 | insomnia:ti OR agrypnia:ti OR hyposomnia:ti OR sleeplessness:ti OR somnolence:ti |
| #2 | 'somnolence'/de |
| #1 | 'insomnia'/exp |

AB, abstract (free text terms appearing in abstracts of articles); DE, descriptor (indexed keywords (subject headings) such as Emtree terms); EXP, explodes controlled vocabulary terms; KW, author keywords (free text terms provided by authors); LIM, limit; MJ, major term (subject heading); TI, title (free text terms appearing in titles of articles).

**Table S2. Summary of quality assessment**

|  | Was randomization carried out appropriately? | Was the concealment of treatment allocation adequate? | Were the groups similar at the outset of the study in terms of prognostic factors? | Were the care providers, participants and outcome assessors blind to treatment allocation? | Were there any unexpected imbalances in drop-outs between groups? | Is there any evidence to suggest that the authors measured more outcomes than they reported? | Did the analysis include an intention-to-treat analysis? If so, was this appropriate and were appropriate methods used to account for missing data? | Did the authors of the study publication declare any conflicts of interest |
| --- | --- | --- | --- | --- | --- | --- | --- | --- |
| Leufkens 2009a ^32^ | Yes | Yes | Yes | Yes^a^ | No | No | Yes, Yes, Yes | Yes^b^ |
| Leufkens 2009b ^33^ | Yes | Yes | Yes | Yes^a^ | No | No | Yes, Yes, NA | No |
| Leufkens 2014 ^31^ | Unclear | Yes | Yes | Yes^a^ | No | No | Yes, Yes, NA | No |
| Mets 2011 ^34^ | Yes | Yes | Yes | Yes^a^ | No | No | Yes, Yes, NA | Yes^b^ |
| O'Hanlon 1984 ^16^ | Unclear | Yes | Yes | Yes^a^ | No | No | Yes, Yes, NA | Not stated |
| O'Hanlon 1984b ^35^ | Unclear | Yes | Yes | Yes^a^ | No | No | Yes, Yes, NA | Not stated |
| O'Hanlon 1986 ^36^ | Unclear | No | Yes | Yes^a^ | No | No | No | Not stated |
| Ramaekers 2011 ^37^ | Yes | Yes | Yes | Yes^a^ | No | No | Yes, Yes, Yes | Yes^b^ |
| Vermeeren 1995 ^38^ | Unclear | Yes | Yes | Yes^a^ | No | No | No | Not stated |
| Vermeeren 1998 ^39^ | Yes | Yes | Yes | Yes^a^ | No | No | Unclear | Yes^b^ |
| Vermeeren 2002 ^40^ | Yes | Yes | Yes | Yes^a^ | No | No | Yes, Yes, NA | Yes^b^ |
| Vermeeren 2015 ^41^ | Yes | Yes | Yes | Yes^a^ | No | No | Yes, Yes, NA | Yes^b^ |
| Vermeeren 2016 ^42^ | Yes | Yes | Yes | Yes^a^ | No | No | Yes, Yes, NA | Yes^b^ |
| Vermeeren 2019 ^27^ | Yes | Yes | Yes | Yes^a^ | No | No | Yes, Yes, NA | Yes^b^ |

NA, not applicable (no missing data).

^a^ Double-blind study, but no further details were provided.

^b^ Study supported by pharmaceutical industry
